# The application of artificial intelligence in the context of person-centred care – a discourse on pitfalls and possibilities

**DOI:** 10.3389/frhs.2026.1796648

**Published:** 2026-05-21

**Authors:** Birgit Schönfelder, Ian Cleland, Tanya McCance, Hanna Mayer

**Affiliations:** 1 Department of Nursing Science, University of Vienna, Vienna, Austria; 2 Department of Nursing Science with Focus on Person-Centred Care Research, Faculty of Health Sciences, Karl Landsteiner University, Krems, Austria; 3 Faculty of Health, University of Applied Sciences Wiener Neustadt, Wiener Neustadt, Austria; 4 School of Computing, Ulster University, Belfast, Northern Ireland, United Kingdom; 5 Institute of Nursing and Health Research, School of Nursing and Paramedic Sciences, Ulster University, Belfast, Northern Ireland, United Kingdom

**Keywords:** AI literacy, artificial intelligence, digital health, nursing, person-centred care, technology-assisted care

## Abstract

Recently, there has been a rise in literature about Artificial Intelligence (AI) in nursing, outlining often positive implications for nursing practice. However, questions remain regarding the compatibility of AI with the fundamental values of nursing care, particularly given the current state and rapid development of AI. In this paper, AI is used as an umbrella term for algorithms that calculate probabilities, by identifying patterns in large data sets, where individual data points have little relevance. This contrasts sharply with nursing, where a person is highly relevant, even more when following a person-centred approach. In this paper, we discuss the conditions under which AI can be used to support person-centred nursing practice, and potentially relevant pitfalls. The paper focuses on the values of person-centred nursing and examines the application of AI in nursing and emphasizes nurses’ ethical values, reflexivity, and ability to advocate for their patients. We argue that AI can support person-centred care in meaningful ways, provided that its use is guided by a critical awareness of its limitations and value implications. We conclude that the development of an advanced, person-centred value-based AI literacy among nurses and healthcare decision-makers is essential to cultivates person-centred practices. Such literacy integrates technical understanding with person-centred values and supports the cultivation of reflective, ethically grounded and person-centred nursing practices. We suggest that nurses equipped with advanced AI literacy, based on and enriched by person-centred values, will be enabled through expanded professional competence to effectively preserve person-centred processes and outcomes when integrating AI tools. Additionally, the balance between integrating evolving technologies and upholding the values of person-centredness requires constant and critical debate within the profession and at a philosophical level.

## 1 Introduction

Recently, there has been a rise in literature about Artificial Intelligence (AI) in nursing, outlining often positive implications for nursing practice. At the same time, potential drawbacks of digital healthcare have been convincingly outlined, such as being forced to choose efficiency over the ability to spend more time with the patient in order to be able to build healthful relationships (1). Further, it was asserted that the present state of digitalization in healthcare does not adequately prioritize respect for persons or their well-being (1). These concerns are directly relevant to the current wave of AI-enabled tools entering care settings.

We take it as given that AI is being adopted in nursing care. In view of the present state of development of AI, a critical question arises as to the extent of its compatibility with fundamental values of nursing care. Person-centred care is the key element of professional nursing practice and reflects its core values. The fundamental elements of person-centred care are healthful relationships among healthcare staff, as well as between nurses, patients, and relatives, mutual respect, and a holistic approach. These elements contribute to the co-creation of knowledge about the individual patient and their needs, thereby supporting patient participation and active involvement in the healthcare process.

Occasionally, the capabilities of AI are being exaggerated, which consequently results in an underestimation of its limitations. Currently, people often associate AI with Large Language Models such as ChatGPT, but this is only one possible application of AI. AI, in a broader sense, calculates probabilities based on data by algorithms so that predictions can be made.

In this paper, AI refers to rapidly evolving data-driven computational systems that generate outputs (e.g., predictions, classifications, or recommendations) by learning patterns from large datasets and mimic human intelligence. These systems are used to support tasks such as diagnosis or patient monitoring, in decision-making or individualized care planning. While AI can process large volumes of information efficiently, we acknowledge that its outputs depend on the quality and representativeness of the data provided (2–4), and on deliberate efforts to mitigate bias (5, 6).

While in some AI applications, a single observation can be extremely important, the mostly minor significance of individual data points in large datasets contrasts sharply with nursing, as for a nurse, the patient is highly relevant, especially when following a person-centred approach. In person-centredness, a person is at the center of all processes and endeavors in healthcare, with the priority being how the person experiences healthcare (7). Person-centredness is defined by McCormack and McCance (8) as:

“an approach to practice established through the formation and fostering of healthful relationships between all care providers, service users and others significant to them in their lives. It is underpinned by values of respect for persons, individual right to self-determination, mutual respect and understanding. It is enabled by cultures of empowerment that foster continuous approaches to practice development”.

To recognize someone as a person is central to person-centredness and represents the most significant contradiction to AI. This aligns with the perceived incompatibility between ‘person-centredness’ and ‘technology’, where person-centredness is associated with closeness and warmth and technology with distance and coldness (9). Further, it is recommended to consider which results are desirable for the persons involved instead of focusing on what could be done from a technical perspective (10), as well as whether innovations are person-centred and if personhood is enhanced (11).

We believe it is important to take a differentiated view of this palpable tension between AI and person-centred care. It is crucial for the profession to find a way to navigate this tension in a self-determined manner. This is the only way to recognize the potential and limitations of AI in nursing, uphold the values of person-centred care in daily practice, and subsequently enable nurses to confidently shape the development of AI.

Given that AI is increasingly integrated into nursing practice, it seems essential to explore whether there are ways to combine the application of AI with the core values of person-centred care. To the best of our knowledge, this combination has not yet been the focus of research. In this paper, we want to demonstrate areas in which the application of AI and the core values of person-centred nursing care are compatible, if nurses have a sound understanding of AI, alongside a person-centred understanding of values. We discuss how and under what conditions AI can be used to support person-centred nursing practice, or rather, what pitfalls need to be considered. Provided that nurses understand how AI works, what it can and cannot do, and are able to reflect on the possibilities and challenges AI poses in specific situations. Such a comprehensive understanding is referred to as AI literacy, which is defined as: “*a set of competencies that enables individuals to critically evaluate AI technologies; communicate and collaborate effectively with AI; and use AI as a tool online, at home, and in the workplace*”. This definition translates in a conceptual framework containing competencies and design considerations for general AI literacy (12). We hypothesize that nurses equipped with advanced AI literacy, based on and enriched by person-centred values, will be enabled through expanded professional competence and clarity of beliefs and values to more effectively preserve person-centred processes and outcomes when integrating AI tools, compared to those possessing solely technical AI literacy.

This paper was inspired by discourse in the community surrounding the tension between two positions that appear to be in direct opposition. On the one hand, there is a commitment to safeguarding the fundamental elements of nursing that are consistent and essential to the profession. On the other hand, there is an openness to new technologies that are developing rapidly. Therefore, this paper, which was informed by literature, and critically discusses the use of AI in nursing practice, based on the values of person-centredness.

## 2 What matters in person-centred care - AIs opportunities and challenges

The concept of “person” in person-centredness encompasses understanding a person's values and beliefs to enable them to be truly authentic and autonomous. Further, the person involved is the patient, their relatives and dear ones, as well as the nurses and healthcare staff. Therefore, delivering and receiving person-centred care, where everyone involved is recognized as a person, leads to a positive care experience as the outcome of person-centred nursing (7). It is important to note that, in person-centredness, the outcome focuses not solely on functionality, but rather on the well-being of all persons involved (1).

A person-centred approach encourages nurses to continuously develop their practice (13). Practice development focuses on the development of knowledge and skills of nurses (14) and is characterized by a critical reflective approach (15). It is important that nurses are able to reflect on their nursing practice critically, to achieve this depth of reflection a nurse has to know themselves and has to have clarity of their own beliefs and values (16). Practice development is enabled by a culture of empowerment and reflects “*healthful workplace cultures that enable everyone to flourish and reach their full potential*” (7). The workplace culture impacts the implementation of person-centred nursing significantly (13). The focus on the transformation of workplace culture and the context in which nursing care is delivered (14) enables nurses to actively co-create their work environment (17). Furthermore, the respect for persons, their personhood, is of significant relevance, as it affects practice development (18), particularly when co-creating a care plan in collaboration with the person affected (7).

If a healthful relationship can be built with a patient or their dear ones, a nurse will be able to get more valuable information as it builds on trust and mutual understanding, which will put the nurse in a position to be able to enable the patient to reach their full potential as they are able to engage authentically (19); therefore, a person-centred care plan should reflect the authenticity of the person concerned (7). This is connected to respect for persons, as it means to respect “what really matters” to a person, which goes beyond physiology and cognition to include feelings, desires, and values. What matters for a person is shaped by their moral code, which is formed by both internal factors like feelings, desires, and values, as well as external factors such as society and culture, and it continues to evolve throughout life (20). In order to be able to respect what really matters to the person and to enable the person to embrace their uniqueness and to make self-determined decisions, a professionally competent nurse with developed interpersonal skills would engage authentically to build a healthful relationship to understand the patient's values and beliefs, to be able to provide holistic care in the patient’s best interest and to be able to be aware of how decisions affect patients and their families.

However, sometimes a person is unable to act authentically or make the best decisions for themselves and needs help from others (19), such as a nurse, to advocate on their behalf during the many decisions made in the care process. Nurses advocate for their patients when they ensure that the person gets the care they need and understands available options (21). This aligns with decisions in person-centred nursing, as those are based on mutual respect and understanding between patients and nurses to enable informed shared decision-making in the patients’ best interests.

With this in mind, we will discuss the opportunities and challenges of applying AI to person-centred care, based on the values of person-centredness: respect for persons, the individual's right to self-determination, and mutual respect and understanding, which are deeply ingrained in the professional ethos of a nurse. These values are facilitated by cultures of empowerment and foster healthful relationships in nursing practice (7).

### 2.1 Healthful relationships

The ability to establish healthful relationships with patients is an essential part of nursing practice and a prerequisite for the successful implementation of AI. This ability is seen as indispensable to being human and the nursing profession. In a study examining the facilitators and barriers to the adoption of AI in nursing, participating nurses insisted that AI, regardless of its task, must align with the core principles of nursing practice, which they listed as empathy, compassion, and the ability to individualize care (22). This aligns with the results of another study, where nurses mentioned that AI could not replace empathy or achieve the necessary emotional intelligence (23). Additionally, nurses emphasized the importance of healthful relationships (24–26) and advocating for their patients’ need for human connection and communication, in order to avoid detachment (24).

In another study by Alruwaili, Alshammari (27), the experience of nurses with AI was reported, and a nurse emphasized that humanity and a general professional attitude guide the usage of AI. Other insights of this study were that AI won't replace nurses, as the nurse's ability to interpret and respond appropriately or to educate and explain AI and how it supports clinical decision-making to patients, is essential to build trust. One of the main findings by Karnehed, Larsson (28) was that establishing a healthful relationship is central to nursing care, as demonstrated by their study of wound care. The participants noted that the trust and continuity of care provided by nurses to treat wounds effectively can’t be replicated by AI. However, AI could, for example, support documentation or other repetitive tasks and therefore make time available for nurses to spend with their patients to deepen their relationship (23, 25, 26, 28).

Ramadan, Alruwaili (22) made another observation: the participating nurses saw AI as a valuable tool to support them, but as unable to replace their interpersonal skills. Paradoxically, AI could provide an opportunity to highlight implicit patterns in nurse-patient communication and make them visible, as shown in a study examining nurses’ language in patient interactions and patients’ speech. The study focuses on patterns in speech to recognize early signs of cognitive decline, which proved to be promising as nurses adjusted their language and tone during communication with cognitively impaired patients (29). This change might sometimes not be explicit, rather than implicit, adapting to the patient's needs and the advantage of AI to detect patterns in data could support nurses and healthcare professionals to identify this patient group early and to make implicit insights explicit. Groeneveld, van Os-Medendorp (30) propose a different approach by suggesting a specialization for nurses in data analysis or engineering to process the data generated by AI and focus on technical aspects, enabling fellow nurses to focus on the patient.

Further, a team of nurses who have healthful and effective relationships with each other, where different personalities and skills are respected and valued, will be able to support and engage with each other in order to develop their nursing practice constantly (31). This will be supported by a psychologically safe work environment, enabling every person involved to flourish (7). The benefits of multidisciplinary teams and a close collaboration with other professionals, which included healthcare as well as tech-related professions, to obtain a comprehensive picture of the patient from different professional perspectives, have been proven to contribute positively to patient care (28).

AI can support the formation and maintenance of healthful relationships if used for example to reduce administrative burden, make implicit patterns in communication visible, or free time for nurses to engage meaningfully with patients and their families. However, AI risks undermining healthful relationships when efficiency-driven implementation displaces relational work, when interpersonal skills are devalued, or when technology mediates interactions in ways that reduce human presence, empathy, and trust (Table 1).

**Table 1:** Key influences on developing healthful relationships.

Opportunities	Risks	Safeguards	Associated technical AI literacy overarching themes (12)	Examples/recommendations
Formation and maintenance of healthful relationships with patients supported by AI	• relational work displaced by efficiency • human presence, empathy, and trust reduced by technology-shaped interaction	• AI aligns with the core principles of nursing practice • a general professional attitude guides the usage of AI	• What is AI? • What can AI do? • How should AI be used?	• reduce administrative burden & free time for nurses to engage meaningfully with patients and their families (23, 25, 26, 28) • make implicit patterns in communication visible (29)
Formation and maintenance of healthful relationships with co-workers supported by AI		• practice development is supported • a psychologically safe work environment is provided		• multidisciplinary teams and a close collaboration with other professionals (28) • technical specialization for nurses (30)

### 2.2 Respect for persons

In recent studies, participants took the initiative to safeguard the patient’s best interests by raising concerns about privacy and the prevention of misuse of data (22, 23, 25, 26) because a data leak or misuse of data would most likely undermine the patients’ trust and negatively affect authenticity, counteracting a person-centred approach. Alruwaili, Alshammari (27) highlighted that the black-box effect of AI could pose challenges if AI is intended to assist in decision-making. The black-box effect of AI describes the inability to understand the reasoning behind recommendations generated by AI. Hoelscher and Pugh (6) agreed and additionally noted that misleading or incorrect output of AI through hallucinations or confabulations, where AI generates plausible information itself, affects the decision-making of healthcare professionals. Without an understanding of how a recommendation is made, it is impossible to verify whether it is based on facts from the patient history in the health record or whether it has been hallucinated by the AI. Consequently, it would be challenging, if not impossible, for a nurse to provide holistic care in the patient's best interests based on AI recommendations. Rony, Numan (24) highlighted the participating nurses’ awareness to make decisions based on their values and beliefs when considering AI recommendations for their patients’ care plan, and their commitment to act as advocates for their patients and to promote trust and fairness. Another example of advocacy behaviors was shown by insisting on a balance between innovation and ethical issues by participating nurses in order to preserve trust in the profession among persons receiving care (25).

Sometimes, a nurse will be challenged to find creative or innovative solutions to be able to provide holistic care in the patient’s best interests. Atalla, El-Gawad Mousa (32) add that the ability to be creative is, next to others, a crucial characteristic of professional behavior and essential to integrate new technologies in nursing practice. Furthermore, the authors emphasize the potential of AI to promote clinical reasoning and creative problem-solving by assisting with administrative tasks and documentation, thereby freeing up cognitive space for creativity. The nurses participating in this study demonstrated a critically reflective approach, recognizing the potential of creativity, if it is evidence-based.

The nurses’ ability to be creative and innovative contrasts with AI, which is often referred to as a mirror, because it only reflects what is already in the dataset, and it is (at least momentarily) unable to create something new or be innovative and creative itself. AI, as also concluded by McCormack (1), depends on a person's ability to program and shape it. Nurses are in a position and are equipped to shape AI development. A nurse is the person who spends a lot of time with the patient and is equipped with professional knowledge, interpersonal skills, and moral integrity to take ethical considerations into account and advocate for their patients. Additionally, nurses are aware of the benefits and flaws of their work environment. This enables nurses to contribute and engage meaningfully in AI development to initiate the development of tools that support patients and nurses within the care environment and workflow. Further, it is respectful to consider the needs of users, the person affected by the application – nurses and patients alike – rather than creating something based on third-party assumptions.

For example, Lee, Park (33) developed a web application in collaboration with nurses to address the needs of community nurses. The application enables nurses to recommend services tailored to individual patients’ needs from a wide range of options. It was highlighted that it was crucial to meet the nurses’ needs during development and involve them during the evaluation. Atalla, El-Ashry (34) studied nurses’ ethical awareness related to AI and work behavior and concluded that ethical awareness fosters a positive attitude towards AI, increases nurses’ innovative practice, and contributes to their well-being. To make these positive changes possible, the authors recommend cooperation with nurses to ensure that their ideas and inspiration are included. These results align with Ball Dunlap and Michalowski (3) who reasoned that nurses’ insights are valuable in enhancing their workflow and addressing patient safety and privacy concerns in the context of data collection in such sensitive areas as direct patient care.

AI can facilitate respectful interactions in nursing if privacy and data security are prioritized and if it simplifies workflow and supports nurses’ professional judgement. If the output of AI is explainable and aligns with nurses’ values and beliefs, it will increase their trust in AI recommendations, which can be achieved by involving nurses in the development process to demonstrate respect for their expertise and leverage their knowledge and creativity. Whereas AI undermines trust through hallucinations, confabulations and the black-box effect, this can negatively impact the patients’ respect for nurses and respectful interactions (Table 2).

**Table 2:** Key influences on respect for persons.

Opportunities	Risks	Safeguards	Associated technical AI literacy overarching themes (12)	Examples/recommendations
Respectful interactions are facilitated		prioritize privacy & data security to prevent misuse of data	• What is AI? • What can AI do? • How should AI be used?	• simplified workflows (3) • Web-application meets nurses and patients’ needs (33) • Positive impact of ethical awareness (34)
Respect for nurses’ expertise, knowledge and creativity		• Involve nurses in AI development process • Support creativity, reflexivity & ethical awareness	• What is AI? • What can AI do? • How should AI be used? • How does AI work? • How do people perceive AI?	
	Blackbox effect	• aim to develop an explainable AI • balance innovation & ethical issues	• What is AI? • What can AI do? • How does AI work? • How should AI be used?	support of nurses’ professional judgment (24)

### 2.3 Right to self-determination

While Alruwaili, Alshammari (27) outline that the use of AI increases the ability to critically reflect on AI-based suggestions, which leads to an enhancement of professional competence. This occurred because nurses in the study questioned AI recommendations and checked if they missed subtle clues or whether the recommendations aligned with the real-world context (like checking charts or vitals). Others warn that shortcuts by using AI should be avoided, as every AI system has its own limitations, and that it should be avoided to bypass critical thinking and being overly reliant on the suggestions of an AI (35). Due to the novelty of AI, there is a lack of reliable studies to determine the effect of heavy AI usage on essential competencies in nursing such as critical thinking or reflexivity.

AI might have the capacity to assist nurses in supporting patients to exercise their right to self-determination, as it is argued that it has the potential to individualize care, resulting in improved patient outcomes (26, 34). In a study conducted by Rony, Numan (24), participating nurses highlighted AI as a possibility to empower patients, as they felt that AI complements their care as it enables them to tailor their care even more to their patients’ needs and preferences. This aligns with the observation that care plans that included AI recommendations were rated as helpful (33). It is true that AI can analyze large amounts of data and detect patterns in a much shorter timeframe and with greater precision than a human could. This advantage could support patients with chronic conditions (26). There is potential when data is automatically generated and continuously collected by a device, for example, blood sugar levels or telemetric cardiological remote monitoring. Another example could be pain management, as stated by Kumar, Seewal (36), relying on different datasets generated by the patient and professionals. AI has the potential to provide an overview, draw attention to unusual data, and alert patients and professionals. This could support nurses and healthcare professionals to provide an individualized treatment or care plan, content for patient education, and recommendations tailored to the patient's needs and life circumstances, which could consequently support a person-centred care approach. However, the algorithm most likely will rely on additional data added by a person to generate an output that truly supports nurses, as AI lacks direct access to context beyond its inputs and therefore cannot independently verify appropriateness. Even then, there are aspects of our identity that define who we are as a person, which cannot be captured by an algorithm through health records or device-generated data, as argued by McCormack (1). Further, the focus in person-centred care is the person behind the patient (18), and it was recently argued by McCormack (1) that a person never reveals the authentic self all the time and has the ability to grow and develop as a person, therefore cannot be reduced to data displayed in a health record.

AI can support the right to self-determination if its recommendations and care plans align with real-world context and supports critical thinking by drawing attention to anomalies pattern in data. The right to self-determination is undermined if AI does not point out possible incompleteness of the data and recommend the provision of additional data or if nurses rely heavily on AI recommendations (Table 3).

**Table 3:** Key influences on right for self-determination.

Opportunities	Risks	Safeguards	Associated technical AI literacy overarching themes (12)	Examples/recommendations
AI recommendations and care plans align with real-world context	• AI misses to point out possible incompleteness of the data & calls for additional data • nurses rely heavily on AI recommendations	• Nurses must be able to critically reflect AI recommendations • Provide different datasets to cover different aspects	• What is AI? • What can AI do? • How does AI work? • How should AI be used?	• AI enables nurses to tailor their care even more to their patients’ needs and preferences (24, 26, 33, 36) • Enhanced professional competence through the ability to critically reflect on AI-based suggestions (27)
AI capability to analyze large amount of data				
AI supports critical thinking				

### 2.4 Mutual respect and understanding

Many decisions are made throughout the care process, based on mutual respect and understanding between patients and nurses. Nurses have a pivotal role in empowering patients to make decisions independently, while also considering how these decisions may potentially impact the patient and how AI could assist with this task. An example where AI could assist nurses, with an exceptional value for novices, is a study conducted by Seo, Kang (37). In this study a nurse with a long work experience outperformed AI in classification of pressure injuries stages 2 and 3, however in cases where the pressure injury was unstageable or if the deep tissues were affected the AI performed better (37). This aligns with Atalla, El-Gawad Mousa (32) were more experienced nurses showed higher clinical reasoning competencies. The speed at which AI can analyze data was described as an advantage by the participating nurses. Additionally, the potential to assist nurses in identifying deteriorated patients (22, 25) or critical incidents (25) earlier and therefore to ensure patient safety and well-being was identified.

On the other hand, it is crucial to consider that an algorithm has no connection to the world; therefore, it cannot verify whether the output is appropriate to the context. Consequently, it is mandatory to constantly match the context provided by the real world and the output generated by AI, as participating nurses highlighted in previous studies (27). Nurses in a study conducted by Karnehed, Larsson (28) highlighted that even when they focus on the color of a wound, for example, the smell would supplement their judgment and final decision, concluding that this would limit an AI, as this information is not instantly available, even if the color of a picture fed into an algorithm is displayed correctly.

Furthermore, as AI has no connection to the real world, it is heavily reliant on the quantity and quality of the data provided. Previous research has questioned whether AI can even consider the complexities of nursing (22). Nursing is complex, as all kinds of information informs the decision-making process. The decision-making process involves the consideration of various factors, including laboratory and radiological findings, which would be documented in the health record. Additionally, information provided by the patient or their relatives is considered, along with the patient's current physical and mental state, and data obtained by observing the patient. A person-centred nurse's approach is holistic, encompassing the patient in its entirety and involving the patient in decision-making. This perspective enables the nurse to constantly integrate new information into their reasoning and reconsider decisions based on it. This demonstrates the competence and skills of the nurse, but it is disadvantageous for an algorithm, as it is unlikely that all of the information will be documented in the health record. Goncalves, Amaro (38) also highlighted that, ideally, information should be made available simultaneously to the algorithm and healthcare professionals. However, they acknowledged the impossibility of this recommendation, as a reaction to a critical incident would be prioritized over entering data into a system. Hoelscher and Pugh (6) use vital signs as an example to explain two further reasons why real-time AI analysis is often limited: firstly, not all systems might be connected to the algorithm, and secondly, vital signs are not only collected via monitoring but also directly from the patient. Furthermore, in nursing, not all patients are the same, and therefore, not only does the available information differ from patient to patient, but the meaningfulness of the information also varies. This contrasts with AI, where data is ideally structured logically, and a dataset would seamlessly contain meaningful information. Consequently, it cannot reason through the decision-making process as a nurse would.

AI can support decision-making if high-quality data is provided, which it can evaluate at an unrivalled speed and thus contribute to patient safety. Decisions based on mutual respect and understanding can be undermined if AI ability to provide recommendations is limited by the inability to consider the inherent complexity of nursing care (Table 4).

**Table 4:** Key influences on mutual respect and understanding.

Opportunities	Risks	Safeguards	Associated technical ai literacy overarching themes (12)	Examples/recommendations
AI enhances patient safety	AI is unable to recognize the complexity of nursing	• High-quality data is provided • Nurses are enabled to constantly compare the real-world context provided and the AI output • Nurses’ decision-making relies not exclusively on AI recommendations	• What is AI? • What can AI do? • How should AI be used?	• AI assists classifying pressure injuries (37), identifying deteriorated patients (22, 25) and critical incidents (25) • Real-time AI analyses limitations outlined (6)
	AI has no connection to the real world			

### 2.5 Cultures of empowerment

A supportive organization system promotes initiative and creativity and ensures freedom and the safety of all persons (13). For example, it would promote transparency and equity during AI implementation through a transparent documentation of AI decisions and regular audits to enable bias detection (39). Professional autonomy and accountability could be addressed through AI polices which provide a structure for responding to AI alerts, ensuring consistent decisions if they are based on AI output (27). The professional autonomy of nurses must be maintained through a supporting work environment that supports continuous learning (27, 40), where nurses are enabled to reflect on AI critically (27).

The possibility of being held accountable professionally is also linked to the potential for innovation and risk-taking, where the best available evidence meets professional judgement and patient/family preferences (13). This contrasts with AI, as AI is not a person and therefore cannot be held accountable. Therefore, a nurse must be able to override an AI-made decision if this decision does not align with the nurse's professional judgment or the preferences of the persons concerned. Following that argumentation, it must be avoided that nurses depend on or adapt to AI systems, for example, through feeling the pressure to develop workarounds, as also mentioned by Alruwaili, Alshammari (27), as the nurses’ professional judgment might be compromised through the need to adapt to an AI system, which won’t work properly otherwise. Power sharing calls for non-hierarchical relationships between those in positions of leadership and staff, working together to achieve goals through agreed values (13). Persons in positions of leadership who adopt a positive and active position towards AI enable an innovative and trustworthy workplace culture where nurses will embrace AI (22). It is essential to establish shared goals and values at the beginning of the process, as they are the foundation and are developed further collaboratively.

The physical environment encompasses design, privacy, and safety as well as operational performance and outcomes (13). AI systems are software-based tools that can function independently of a physical environment. However, it potentially affects or is affected by the physical environment. As the infrastructure provided has a strong impact on AI implementation (22, 27), this includes cost-effectiveness and data security (40), reliable systems and stable internet connections to avoid data loss through glitches of weak internet connections, as well as adequate nurse staffing (27). Implementing AI means that nurses need to learn how to navigate the software and to integrate it into their workflow, which was mentioned as overwhelming in general and even more with a high workload caused by inadequate staffing (26). Furthermore, there is a risk that AI does not deliver what it promised and is not able to increase efficiency, instead increasing administrative tasks (28), which could necessitate double documentation to prevent data loss or re-entry of data due to glitches, which leads to a decreased time a nurse is able to spend with the patient. Additionally, El-Sayed, Alsenany (39) raised privacy concerns from a nurse’s perspective, if staff's personal data is analyzed by an algorithm without consent.

A person-centred workplace culture taking the issues mentioned into account would build trust in the AI and the output generated. This could most likely lead to a psychologically safe work environment where nurses would embrace AI as they would feel safe to employ it.

AI can support cultures of empowerment if the appropriate infrastructure, policies and training programs are in place, and if particular importance is given to privacy and potential bias. AI undermines cultures of empowerment if it creates an additional workload and forces nurses to accept AI decisions that contradict their experience and knowledge (Table 5).

**Table 5:** Key influences of cultures of empowerment.

Opportunities	Risks	Safeguards	Associated technical ai literacy overarching themes (12)	Examples/recommendations
AI supports cultures of empowerment		• promote initiative, creativity and safety • ensure consistent AI supported decisions • provide appropriate infrastructure, policies, training programs and adequate staffing • Nurses must be able to override AI decision/not follow AI recommendations	• What is AI? • What can AI do? • How should AI be used? • How do people perceive AI?	• transparency and equity through transparent documentation of AI decisions and regular audits to enable bias detection (39) • AI polices providing structured responses to AI alerts (27) • Maintenance of nurses’ professional autonomy through continuous learning (27, 40) • Enable nurses to critically reflect AI (27) • Avoid nurses adapting to AI or developing workarounds (27) • Avoid additional workload for nurses (28) • Address privacy concerns adequately (39)
	• Additional workload • Nurses forced to accept AI decision • Privacy issues			

## 3 Discussion

**Figure 1:**
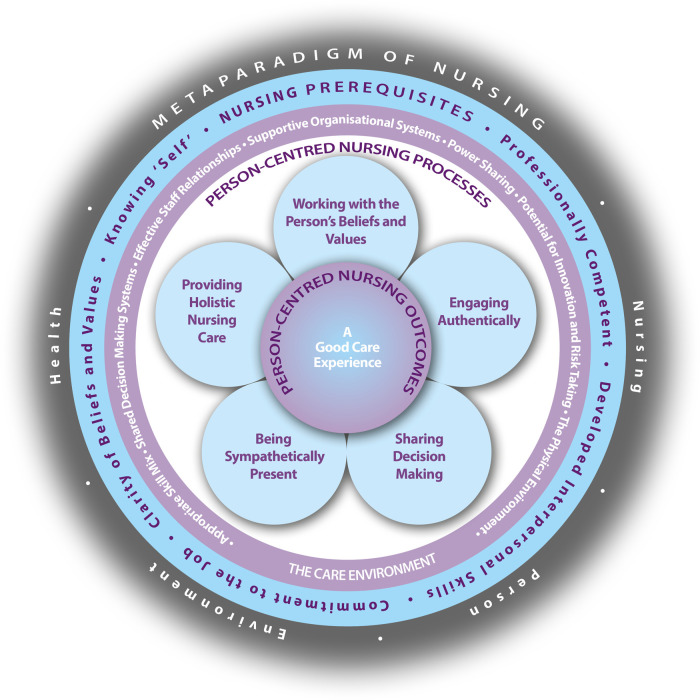
Person-centred nursing framework (7).

Through the discussion in this paper, we demonstrate that AI is increasingly integrated into nursing care, as the growing body of literature suggests. Whereby the estimated number of AI applications that have not been scientifically monitored and therefore published is likely to be much higher. Furthermore, the application of AI and person-centred care is compatible to a certain extent, provided advanced AI literacy that is informed by and enriched by person-centred values, thereby becoming an additional skill for nurses. This advanced AI literacy could enable nurses to critically reflect on the opportunities and challenges of AI while considering fundamental values of nursing. As they are enabled through expanded professional competence and clarity of beliefs and values to effectively preserve person-centred processes and outcomes when integrating AI tools in their nursing practice. These insights imply that these competencies are embedded within the prerequisites of the person-centred nursing framework (PCNF) (Figure 1).

To the best of our knowledge, this is the first paper to critically discuss the extent to which AI can be aligned with person-centred values in nursing practice. Further empirical investigation is required to test our hypothesis, and a competency framework needs to be refined in future research. Therefore, as this represents an initial exploration of the topic, it is not yet possible to specify implications for nursing practice.

Once the competency framework has been established, PCNF might offer guidance on implications for nursing practice. The PCNF can be used to exemplify key elements of person-centredness in nursing practice in a comprehensible and meaningful way. It encompasses the comprehensive knowledge and skills of nurses to be able to provide person-centred care. It stipulates requirements for a person-centred care environment, thereby empowering nurses with the appropriate attitude and knowledge to provide person-centred care through the person-centred processes in order to achieve a good care experience for every person involved (7).

The PCNF is embedded in the metaparadigm of nursing. This paper focused on the concept of “person” within the metaparadigm of nursing, as it emphasizes the importance of the values of person-centred nursing and represents the most significant contradiction to AI. The concept of person encompasses understanding a person's values and beliefs to enable them to be truly authentic and autonomous across the modes of being. The modes of being considered that a person develops relationships (being in relation) and lives in a society rather than being isolated (being in a social world) - both shaping a person’s values and beliefs. Further, it is essential to know one's values and beliefs (being with self) and consider the care environment that affects the context in which care is provided (being in place) (7). The modes of being apply to every person involved (7), offering insights from different perspectives on the practical implementation of person-centred care (13). This entails that the perspectives of patients, their dear ones, and healthcare professionals are considered.

A person-centred value-based AI literacy enables nurses to uphold fundamental person-centred values when engaging with AI and to further develop their practice. AI literacy in general is critical for a safe and effective application, as it enables a person to interpret AI's output, recognize limitations, and promote awareness of cognitive bias in order to be able to apply AI in daily nursing practice and derive conclusions. Groeneveld, van Os-Medendorp (30) suggested that nurses could achieve different levels of AI competency, ranging from general to expert, depending on their specialization. AI literacy is influenced by the nurses’ attitude and anxiety regarding AI (41, 42) and an increased understanding of AI through training (22, 27). AI could support nurses, but nurses must be aware of AI's possibilities, limitations, and risks. A nurse should be aware that bias can occur either in the dataset or in the context of AI usage – for example, automation bias, where the generated outcome is accepted without critical reflection (43). AI literacy is therefore significant in order to be aware of the limitations of AI. For example, it is essential to be aware that the output from the AI system must be manually matched to the actual patients’ condition by the nurse. Therefore, should AI detect abnormal vital signs, the nurse responsible for the patient is required to first check the patient instead of responding automatically to the alarm (27). Additional alarms bear the possibility of further increasing alarm fatigue. Alarm fatigue is defined as “*excessive exposure to the stimulus generated by the monitoring unit*”. It is reported as a burden and leads to disturbances in patient care due to interruptions, as well as reduced trust in the system if alarms are false or insignificant (44).

Another issue affecting AI literacy is the ‘black box’ effect, whereby it is impossible to understand the basis on which recommendations are made; this makes it difficult to evaluate the output of AI (12). However, professionals and patients must be able to evaluate these outputs. One consequence of a person being unable to evaluate the output due to the ‘black box’ effect of the algorithm is that it prevents them from flourishing (39) because it negatively impacts trust when it remains uncertain on what basis decisions are made. Trust is built if nurses are able to educate patients and explain how AI supports their decision-making, as they understand how the recommendation was made and are able to respond appropriately to AI output. Human flourishing is the outcome of person-centredness and is achieved by being enabled to integrate creativity and all different forms of knowledge essential to nursing practice to grow personally and professionally (7). If AI prevents nurses and patients from flourishing, it contradicts a person-centred approach. The framework for Trustworthy AI clearly states that explicability is one of the four ethical principles of the framework. Further, it is explained that if a ‘black box’ effect occurs, other measures, for example, traceability or transparent communication, are required to increase transparency and explainability (45).

Ethical challenges are another main concern regarding the application of AI in nursing. A recent study raised awareness of the importance of equipping nurses with the necessary knowledge and skills to deal with this issue (39) and Atalla, El-Ashry (34) highlighted nurses’ awareness of ethical issues regarding AI usage. Further, ethical considerations are essential in nurses’ decision-making; therefore, ethical viewpoints are mandatory for AI to be able to follow moral principles in such a sensitive area as healthcare and nursing (24). The International Council of Nursing definition of nurses and nursing (46) reflects the code of ethics for nurses (47), which in turn reflects professional nursing standards. Due to the high level of responsibility that our profession entails, ethical aspects are fundamental and accompany nursing staff throughout their entire career and across the full spectrum of their roles. Nurses, therefore, have a deep understanding of and a strong commitment to ethical issues, which puts them in a position to assess the ethics of the application of AI in nursing. This paper highlighted nurses’ sensitivity to ethical issues and the priority they assigned to these aspects. In regard to the regulation of AI, the High-Level Expert Group on AI (AI HLEG) established by the European Commission (45) has delineated a framework for Trustworthy AI to guide the development of AI applications from an ethical perspective, to avoid bias as far as possible. A biased data set has the potential to exacerbate existing imbalances (6, 45), for example, regarding race or gender, or to produce incorrect or faulty outputs (3, 6). To address bias, the data set needs to be of high quality and diverse (34), and documentation in the health record has to be accurate (2). However, a recent systematic review has drawn attention to a fundamental issue by exploring quality criteria for nursing documentation, as it concluded that there is still a lack of evidence-based quality criteria for nursing documentation (48). This could potentially result in adverse consequences if AI is developed based on nursing documentation, as it remains ambiguous what constitutes high-quality nursing documentation. Considering that one of the potential databases for AI, nursing documentation, is currently managed in a heterogeneous manner, it is particularly important that nurses are involved in AI development in order to address ethical issues.

The results highlight that nurses consider their patients’ wishes and their workplace when discussing the benefits and limitations of AI. Therefore, it is important that nurses actively participate in the development of AI solutions, as they are equipped with professional knowledge, interpersonal skills, and moral integrity to consider ethical issues and advocate for their patients. If their voices are heard and valued, this will most likely benefit the discussed AI algorithms, which will subsequently benefit patients and nurses. Therefore, nurses should be encouraged to participate in AI development, and their insights should be sought before implementation so that their voices, concerns, and wishes can be taken into account. Otherwise, nurses will have to adapt to technology (26, 27) developed by third parties. Despite the recommendation to prioritize humanistic aspects of nursing in AI development (23), there is no guarantee that the fundamental essence of nursing, such as healthful relationships or a holistic approach to patients, is adequately addressed. Technical solutions may be found, especially as AI is developing so rapidly. Moreover, it is imperative that developers have a comprehensive understanding of the issues they attempt to address. Additionally, they must be informed of the benefits the algorithm offers from the perspective of person-centred nurses, ensuring that these benefits are not overshadowed during the development process.

The ability to be an authentic person is a fundamental aspect of person-centredness (7). The insights reflected in this paper indicate that the modes of being (7) and fundamental aspects of nursing practice are supported by AI to varying extents. Sometimes AI is only able to assist indirectly, if at all. These include, in particular, the focus on perceived personhood or the formation of healthful relationships. It is important to note that there is no hierarchy between a nurse and a patient; they are both seen as a person (49) with individual needs and desires. However, there is a hierarchy in comparison to AI, which does not represent a person, but an algorithm processing data under human oversight. This is consistent with the Trustworthy AI framework, which identifies human oversight as one of its seven key requirements (45).

We conclude that prerequisites for the successful application of AI in nursing are that nurses adopt a person-centred approach and cultivate advanced AI literacy. This is informed by and enriched by the values of person-centredness, with the aim to ensure appropriate application of AI along the PCNF. Second, the rise of AI in healthcare introduces an additional factor that affects the creation of knowledge in nursing and potentially practice development. Therefore, AI literacy is essential for nurses to be able to effectively manage AI and critically evaluate AI outputs and integrate them into their practice without losing focus on constantly developing their practice in line with person-centred values.

The overarching vision is for AI to support nursing staff, without compromising their autonomy or professionalism, and to enable them to spend more time fostering healthy relationships with patients. It is equally important to consider the working environment, which should encourage person-centred values and person-centred practice development. A supportive work environment would uphold the professional autonomy of nurses and most likely lead to a psychologically safe work environment where nurses would embrace AI as they would feel safe to employ it. Consequently, person-centred value-based AI literacy is also crucial for decision-makers. Educating themselves about AI enables them to explain it adequately to their team, fostering trust, reducing skepticism and contributing to the responsible application of AI to enhance patient care. Provided that all aspects of the PCNF, which covers all issues discussed in this paper within its constructs, are considered.

Therefore, nurses who adopt a person-centred approach deliberately integrate new technologies into their practice, are aware of their appropriate use and ethical implications, are able to consider the limitations of the algorithm, and have an appropriately adjusted workplace; technology won't hinder them from providing person-centred care. Instead, AI could be an additional resource to improve patient outcomes if the algorithm is able to support the relational and ethical core of person-centred care.

Additionally, we would like to suggest that the ongoing discussion about the tension between integrating evolving technologies and upholding the values of person-centredness requires constant and critical debate and must be continued at a philosophical level. Nevertheless, AI is already a reality and is being used in various areas of nursing. In order to employ it in a manner consistent with person-centred care and to be able to assess its impact on person-centredness, an advanced form of AI literacy is essential: person-centred value-based AI literacy. This is predicated on the integration of person-centred values, encompasses the capacity to utilize AI, and cultivates person-centred practices.

## Limitations

The discourse should be pursued by engaging in critical reflection and contemplating the benefits and drawbacks of AI in the context of nursing. The present article has highlighted selected aspects of person-centred nursing and AI without claiming to be exhaustive or systematic. Consequently, a degree of selection bias is unavoidable, and this paper merely offers an initial insight into this matter. Additionally, to address the paper's focus of AI in nursing practice, the included empirical research is on nurses’ experience of AI in nursing practice; therefore, papers focusing on robotics, patient experience, nursing education and nursing leadership were excluded. Moreover, person-centredness and nursing in general should be considered as a multifaceted and complex construct. The interdependent nature of the constructs of the frameworks (7, 50) posed an additional challenge for analysis. It is impossible to separate and discuss person-centred values underpinning the PCNF inter-related of the framework’s constructs, which are already inter-related. This suggests that it is not possible to discuss the effects of AI on a solitary aspect of person-centredness in isolation. Nevertheless, efforts were made to avoid overlap between the values to improve the comprehensibility of the insights.

## Data Availability

The original contributions presented in the study are included in the article/supplementary material, further inquiries can be directed to the corresponding author/s.
